# Medical News

**Published:** 1851-12

**Authors:** 


### MEDICAL NEWS.

Organization of the Kentucky State Medical Society.—The physicians of Kentucky met at Frankfort, in that State, in Convention, on the first of October last, and adopted a constitution for the formation of a State Medical Society. The first annual meeting of the State Medical Society of Kentucky was subsequently held, and the following gentlemen were elected officers for the ensuing year: *President*, Dr. W. L. Sutton, of Georgetown; *Vice Presidents*, Drs. W. S. Chipley, of Lexington, and J. Dudley, of Nicholasville; *Recording Secretary*, Dr. W. C. Sneed, of Frankfort; *Corresponding Secretary*, Dr. R. J. Breckinridge, Jr., of Louisville; *Treasurer*, Dr. R. W. Glass, of Shelbyville; *Librarian*, Dr. B. Monroe, of Frankfort. Various standing committees were appointed, and the city of Louisville was fixed upon for the holding of the next annual meeting of the Society, on the third Wednesday in October, 1852.

Delegates to the National Medical Convention from Chester county, Penna., to meet at Richmond in May next: Doctors A. H. Gaston, W. Worthington, J. B. Brinton, W. D. Hartman and S. Harvey.

Appointment.—Dr. Pereira has been appointed Physician to the London Hospital, in the room of Dr. Frampton.

The Quarantine Congress at Paris.—It is to be hoped that the labors of the eminent men whom some of the European States have sent to Paris, to frame laws and regulations regarding Quarantine, in keeping with the scientific views of the present age, will have a satisfactory result. The congress has been assembled for the last two months, and is composed as follows: Austria, Dr. Meni, chief physician of the province of Dalmatia, author of several esteemed works; Tuscany, Dr. Betti, Professor of Surgery at Florence, and for a long time at the head of the sanitary affairs of the country; Naples, Dr. Carbonaro; England, Dr. Sutherland, whose knowledge of sanitary matters we need not dwell upon; Genoa, Dr. Bô; Greece, Dr. Costi.

Professor Gorini.—This gentleman, who is professor of natural history at the University of Lodi, made lately in London, before a circle of private friends, a very remarkable experiment illustrative of his theory as to the formation of mountains. He melts some substances, known only to himself, in a vessel, and allows the liquid to cool. At first it presents an even surface, but a portion continues to ooze up from beneath, and gradually elevations are formed, until at length ranges and chains of hills are formed, exactly corresponding in shape with those which are found on the earth. Even to the stratification the resemblance is complete, and M. Gorini can produce, on a small scale, the phenomena of volcanoes and earthquakes. He contends, therefore, that the inequalities on the face of the globe are the result of certain materials, first reduced, by the application of heat, to a liquid state, and then allowed gradually to consolidate. In another and more practically useful field of research, the learned professor has developed some very important facts. He has succeeded, to a most surprising extent, in preserving animal matter from decay, without resorting to any known process for that purpose. Specimens are shown by him of portions of the human body which, without any alteration in their natural appearance, have been exposed to the action of the atmosphere for six or seven years; and he states that, at a trifling cost, he can keep meat for any length of time, in such a way that it can be eaten quite fresh. The importance of such a discovery, if on a practical investigation it is found to answer, will be more readily understood when it is remembered that the flocks of sheep in Australia are boiled down into tallow, their flesh being otherwise almost valueless, and that in South America vast herds of cattle are annually slaughtered for the sake of their hides alone.—*Lond. Med. Times.*

Obituary.—Dr. Roe, Deputy Inspector General of Hospitals, Madras, who died there on the 4th of Sept., from the effects of an accident, entered the medical branch of the army in 1808. He served in the battle of Corunna, and in the expedition to Walcheren in 1809. From 1811 to 1814, he served in the Peninsular war, and had received the silver war medal with ten clasps, for his active services on the following occasions: at Corunna, Albuhera, the seiges of Ciudad Rodrigo and Badajos, the battles of Salamanca, Vittoria, Pyrenees, Nivelle, Orthes, and Toulouse.

M. de Savigny, Member of the Academy of Sciences of Paris, has just died at Versailles, at an advanced age. M. de Savigny had acquired much celebrity by his scientific labors during the campaign of Egypt, and by his investigations on the anatomy of insects, crustaceæ, &c.

